# Causal association between inflammatory bowel disease and acute pancreatitis: a two-sample bidirectional mendelian randomization study

**DOI:** 10.3389/fgene.2024.1324893

**Published:** 2024-08-14

**Authors:** Cong Zhang, Xiujing Fan, Zhijun Li, Zongyi Hu, Chengcheng He, Shanping Wang, Mingsong Li

**Affiliations:** ^1^ Guangdong Provincial Key Laboratory of Major Obstetric Diseases, Department of Gastroenterology, Guangdong Provincial Clinical Research Center for Obstetrics and Gynecology, The Third Affiliated Hospital, Guangzhou Medical University, Guangzhou, China; ^2^ Department of Gastroenterology, The First People’s Hospital of Foshan, Foshan, China

**Keywords:** inflammatory bowel disease, acute pancreatitis, mendelian randomization, genome-wide association studies, single-nucleotide polymorphisms

## Abstract

**Background:**

Acute pancreatitis (AP) is an extraintestinal manifestation of inflammatory bowel disease (IBD). Numerous observational studies have reported an increased risk of AP in patients diagnosed with IBD. However, the causal association and directionality between IBD or its subtypes and the development of AP remains unclear due to the limitations of observational research. This study aims to explore the relationship between IBD or its subtypes and AP risk using Mendelian Randomization (MR) method.

**Methods:**

A two-sample bidirectional MR study was conducted, selecting genetic variants associated with IBD and AP as instrumental variables from the International Inflammatory Bowel Disease Genetics Consortium (IIBDGC) and FinnGen databases, respectively. The inverse-variance weighted (IVW) method used as the primary approach for causal inference. The Cochran Q test was employed for heterogeneity assessment. Sensitivity analyses were performed using the MR Egger intercept test, MR-Presso, and Leave-one-out method.

**Results:**

The results revealed that IBD (OR = 1.049, 95% CI = 1.010–1.090, *p* = 0.013) and ulcerative colitis (UC) (OR = 1.057, 95% CI = 1.013–1.102, *p* = 0.011) were significantly associated with an increased risk of AP. However, Crohn’s disease (CD) (OR = 1.023, 95% CI = 0.993-1.055, *p* = 0.134) did not show a causal association with the risk of AP. Interestingly, AP was suggestively associated with a decreased risk of CD (OR = 0.797, 95% CI = 0.637-0.997, *p* = 0.047). Furthermore, there was no causal association between AP and the risk of IBD (OR = 0.886, 95% CI = 0.753-1.042, *p* = 0.144) or UC (OR = 0.947, 95% CI = 0.773-1.159, *p* = 0.595).

**Conclusion:**

In conclusion, this study provides genetic evidence supporting the causal influence of IBD (specifically UC) on AP, while CD does not appear to have a causal impact on AP.

## 1 Introduction

Inflammatory bowel disease (IBD), comprising mainly Crohn’s disease (CD) and Ulcerative colitis (UC), is a chronic and recurring digestive disorder (Agrawal et al., 2022). Previous research has demonstrated that IBD may result from autoimmune overactivation triggered by genetic, environmental, and intestinal microbiota factors (Torres et al., 2017; Ungaro et al., 2017). Additionally, IBD often presents extraintestinal manifestations, including arthritis, uveitis, and skin disorders (Barreiro-de Acosta et al., 2023). Despite significant advancements in IBD treatment brought about by biologics and small molecules, a complete cure remains elusive (Parigi et al., 2023). Consequently, understanding the etiology and outcomes of IBD remains of utmost importance.

Acute pancreatitis (AP) is a frequently encountered abdominal emergency that can be fatal (Petrov and Yadav, 2019). It occurs due to the inappropriate activation of pancreatic digestive enzymes, leading to the self-digestion of pancreatic tissue (Walkowska et al., 2022). Many reviews have summarized that pancreatitis can also manifest as an extraintestinal complication of IBD (Montenegro et al., 2022; Conti Bellocchi et al., 2023; Massironi et al., 2022). The occurrence of AP is about four times more prevalent in individuals with CD compared to the general population, and about twice as high in those with UC (Rasmussen et al., 1999). This suggests that IBD may be one of the contributing factors in the development of AP. However, aside from IBD itself, the occurrence of AP in IBD may also be caused by conditions such as gallstones, medication, duodenitis, and invasive medical procedures (Montenegro et al., 2022). It is important to note that the previous studies have been observational and cannot establish a causal relationship between IBD and AP.

Mendelian randomization (MR) studies enable the inference of causal relationships between exposure and outcomes by utilizing genetic variations as instrumental variables identified in genome-wide association studies (GWAS) (Birney, 2022). Single nucleotide polymorphisms (SNPs) are commonly employed as instrumental variables in MR. Since SNPs are randomly assigned at conception, they are not influenced by confounding factors and reverse causality (Sekula et al., 2016). Moreover, bidirectional MR studies allow for the identification of reciprocal causal relationships between two diseases (Davey Smith and Hemani, 2014). With the rapid development of GWAS-related research and the accessibility of public datasets, two-sample MR studies are increasingly conducted and valuable in elucidating causal relationships among different diseases. A recent MR study investigated the impact of 30 risk factors on pancreatitis and found that IBD may be one of the causes of AP (Mao et al., 2023). However, the study did not analyze the specific subtypes of IBD and their effects on AP. In this study, we use a two-sample bidirectional MR design to comprehensively investigate the causal relationship between IBD or its subtypes and AP, in order to provide new insights for clinical practice.

## 2 Methods

### 2.1 Study design

**FIGURE 1:**
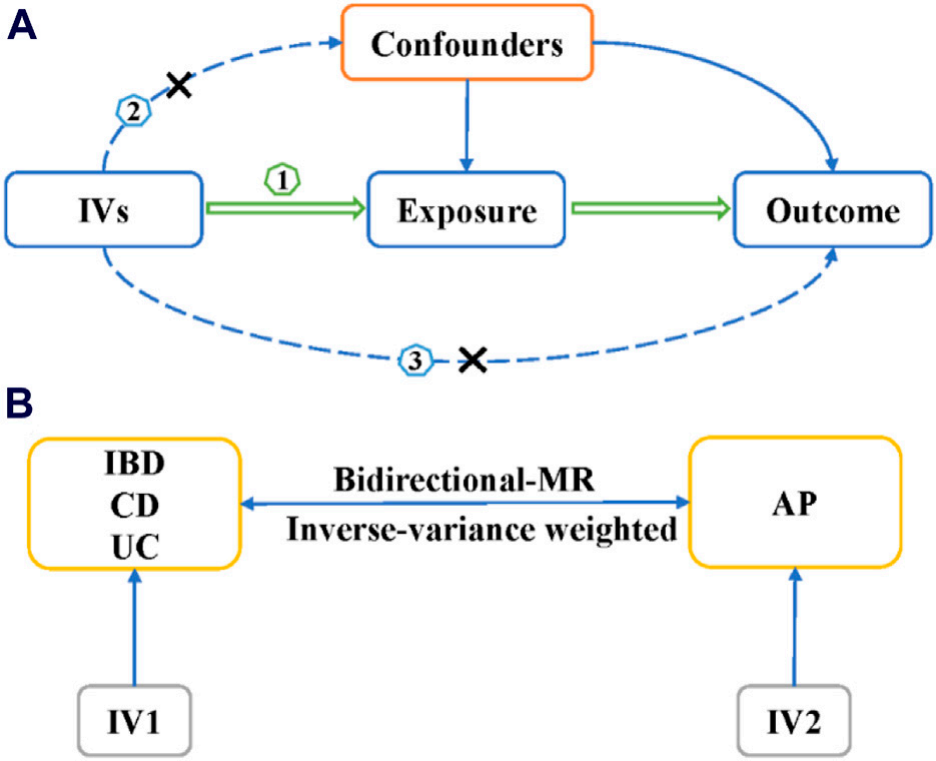
The design of Mendelian randomization study. **(A)** The three basic assumption of MR studies. **(B)** The bi-direction MR design. IVs, instrumental variables; IBD, inflammatory bowel disease; UC, ulcerative colitis; CD, Crohn’s disease; MR, Mendelian randomization; AP, acute pancreatitis.

The aim of this research is to examine the relationship between Inflammatory bowel disease and acute pancreatitis using a two-sample bidirectional MR method. We use multiple single nucleotide polymorphisms (SNPs) as the instrumental variables (IVs) to represent genetic variation associated with exposure factors. For an MR study, it is crucial to satisfy the following three aspects: 1). IVs are directly related to exposure factors; 2). IVs are not associated with other confounding factors influencing the outcome; 3). IVs do not directly affect outcomes but rather influence them through exposure factors (Figure 1A). The inverse-variance weighted (IVW) method serves as the primary approach for evaluating the causal relationship between IBD and its subtypes, as well as AP (Figure 1A). In the bidirectional MR study, IBD or its subtypes are considered as exposure factors when AP is the outcome, and *vice versa* (Figure 1B). The selection of IVs and MR analysis in this study is based on the TwoSampleMR R package (version 0.5.6).

### 2.2 Data sources

The genome-wide association studies (GWAS) data for IBD, including CD and UC, was obtained from the IEU GWAS database, which incorporates data from International Inflammatory Bowel Disease Genetics Consortium (IIBDGC) (Liu et al., 2015). The IBD data consists of 12,882 cases and 21,770 controls (Table 1). Additionally, the CD data includes 5,956 cases and 14,927 controls, while the UC data comprises 6,968 cases and 20,464 controls (Table 1). Diagnosis for all these cases was based on established endoscopic, radiological, and histopathological criteria.

**TABLE 1:** The characteristics of the GWAS summary data.

Phenotype	Population	Case/control	Sample size	nSNP	F value	GWAS ID
Inflammatory bowel disease	European	12882/21770	34652	50	10.043–56.976	ieu-a-31
Crohn’s disease	European	5956/14927	20883	43	10.075–111.506	ieu-a-30
Ulcerative colitis	European	6968/20464	27432	30	10.404–66.336	ieu-a-32
Acute pancreatitis	European	6233/330903	337136	5	10.129–11.949	K11_ACUTPANC

nSNP, the number of instrumental variables.

The GWAS data for AP was obtained from the FinnGen consortium data (R9 release, https://www.finngen.fi/en) (Kurki et al., 2023). The AP data included 6,233 cases and 330,903 controls (Table 1). Only individuals of European descent were included as cases or controls in this study. As our study involves the reanalysis of publicly available data, no additional ethical approval is required.

### 2.3 Selection of genetic instrumental variables

The selection of IVs strictly adheres to the three fundamental assumptions of MR research mentioned above. When IBD or its subtypes are used as exposure factors, we screened IVs using the GWAS data of IBD or its subtypes. Firstly, SNPs were filtered using a strict *p*-value cutoff (*p* < 5 × 10^−8^), and SNPs in linkage disequilibrium (*r* ^2^ > 0.01, genomic region <10,000 KB) were removed. Secondly, we computed the F-value to assess the strength of SNPs, and SNPs with F-value ≤10 were deemed weak and excluded. The F-value formula is F = ((N-k-1)/k) × (*R* ^2^/(1-R^2^)), where N represents the sample size in GWAS analysis, K represents the number of IVs, and R indicates the extent to which IVs account for exposure factors. Thirdly, we merged data related to exposure factors with the SNP data related to outcomes and utilized the LDproxy tool on the LDlink website (https://ldlink.nci.nih.gov/?tab = ldproxy) to identify proxy SNPs for SNPs absent in the outcome GWAS data (Myers et al., 2020). Moreover, to prevent direct correlation between the IVs and outcome, we exclusively selected SNPs with exposure *p*-value < outcome *p*-value. Finally, we removed SNPs related to confounding factors (such as smoking, alcohol, education level, triglycerides, and cholelithiasis) that may impact outcomes, based on data from the PhenoScanner website (http://www.phenoscanner.medschl.cam.ac.uk/), and obtained the final set of SNPs (Kamat et al., 2019). Additionally, we excluded the SNP rs2647087, as it has been reported in the literature to be associated with thiopurine-induced AP (Heap et al., 2014). When AP is used as exposure factors and IBD or its subtypes as the outcome, we employed a truncation value of *p* < 5 × 10^−6^ due to the inability to filter SNPs using a threshold of *p* < 5 × 10^−8^. In addition, the other procedure is the same as above except that we did not use PhenoScanner to screen SNPs impacting outcomes.

### 2.4 MR analysis

We mainly employed the fixed-effect inverse-variance weighted (IVW) method as the primary approach to estimate the relationship between IBD or its subtypes and AP. Additionally, we also employed MR Egger and Weighted median methods to assist in analyzing the IVW results. Moreover, given that the IVW analysis is sensitive to outlier and horizontal pleiotropy, we conducted three sensitivity analyses (MR Egger intercept, Leave-one-out, and MR-PRESSO methods) to ensure result consistency. The MR Egger method employs the intercept obtained from regression analysis to examine the horizontal pleiotropy. The “Leave-one-out” method involves sequentially excluding each SNP to calculate the meta-effect of the remaining SNPs and identify outliers. The MR-PRESSO distortion test was utilized to detect potential outliers causing horizontal pleiotropy, and the IVW results were further adjusted by removing these outliers. The Cochran Q test was used to evaluate the degree of heterogeneity among IVs. In this study, heterogeneity is not significant (*p* > 0.05), so we employed a fixed-effects model of IVW.

### 2.5 Statistics

Results are reported in terms of odds ratio (OR) of the 95% confidence interval (CI). The corrected bilateral *p* < 0.05/3 was considered statistically significant after Bonferroni correction, while *p* values ranging from 0.05/3 to 0.05 were regarded as suggestive significance. All analyses were performed based on R software (version 4.3.0). The R packages used include data. table (version 1.14.8), R.utils (version 2.12.2), TwoSampleMR (version 0.5.6), and MR-PRESSO (version 1.0) (Ong and MacGregor, 2019).

## 3 Results

### 3.1 Genetic IVs for IBD

After a strict screening process, we identified 50, 43, and 30 IVs in IBD, CD, and UC, respectively (Table 1, Supplementary Tables S1–S3). All the F-value for these IVs are greater than 10 (Table 1). The *p*-value for the Cochran Q test of IBD, CD, and UC studies are 0.458, 0.532, and 0.811, respectively (Table 2). Therefore, we used the fixed-effect model of IVW to evaluate the causal effects of IBD or its subtypes on AP.

**TABLE 2:** The sensitivity analyses for MR study.

Exposure	Outcome	nSNP	Heterogeneity test		MR egger pleiotropy test		MR-PRESSO global outlier test
			Q	*p*-value	Intercept	*p*-value	Outlier
IBD	AP	50	49.373	0.458	−0.00055	0.952	None
CD	AP	43	40.600	0.532	−0.00174	0.830	None
UC	AP	30	22.227	0.811	−0.00295	0.828	None
AP	IBD	5	0.362	0.985	0.00780	0.754	None
AP	CD	5	2.785	0.594	0.01910	0.584	None
AP	UC	5	2.624	0.623	−0.01237	0.692	None

IBD, inflammatory bowel disease; UC, ulcerative colitis; CD, Crohn’s disease; MR, Mendelian randomization; AP, acute pancreatitis; nSNP, the number of instrumental variables.

### 3.2 Causal effects of IBD or its subtypes on acute pancreatitis

**FIGURE 2:**
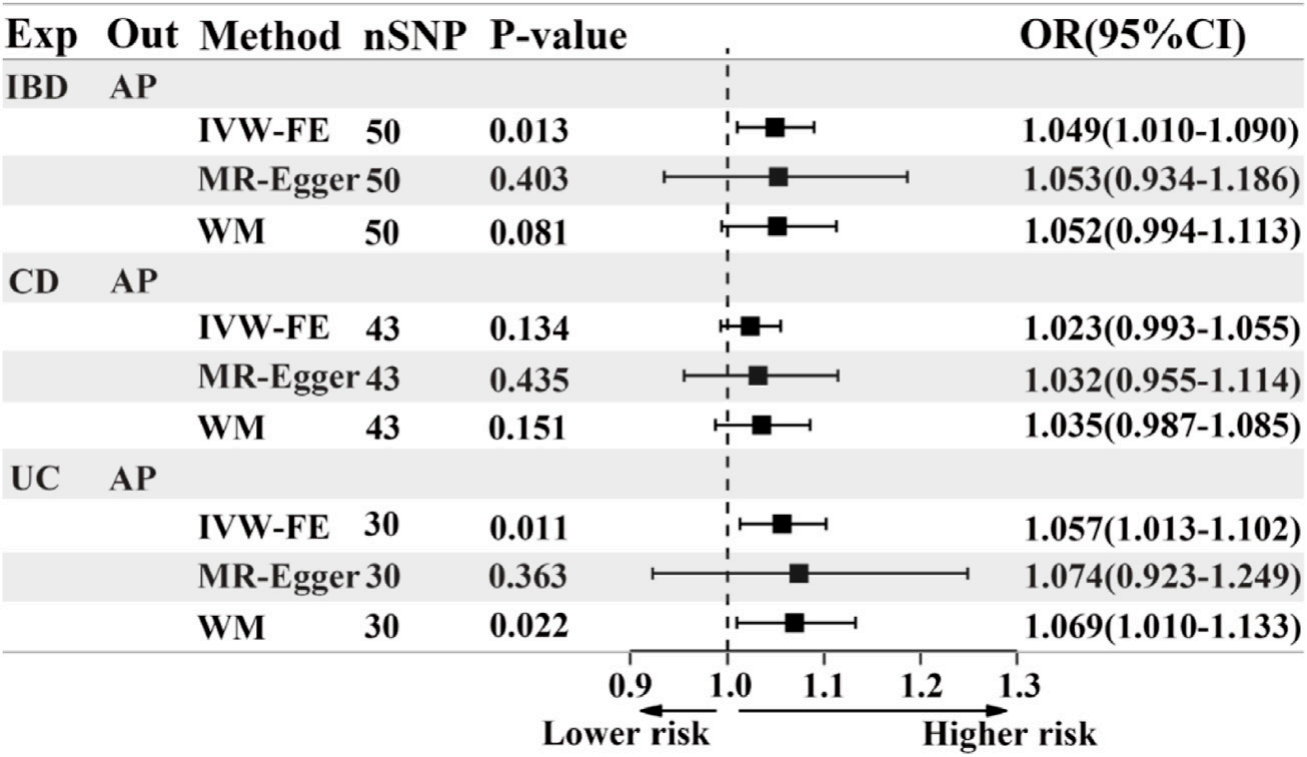
The forest plot of MR results when IBD or its subtypes are the exposure and AP is the outcome. Exp, exposure; Out, outcome; IBD, inflammatory bowel disease; UC, ulcerative colitis; CD, Crohn’s disease; AP, acute pancreatitis; nSNP, the number of instrumental variables, OR, odds ratio; 95%CI, 95% confidence interval; IVW-FE, Inverse-variance weighted (fixed effects); WM, Weighted median.

**FIGURE 3:**
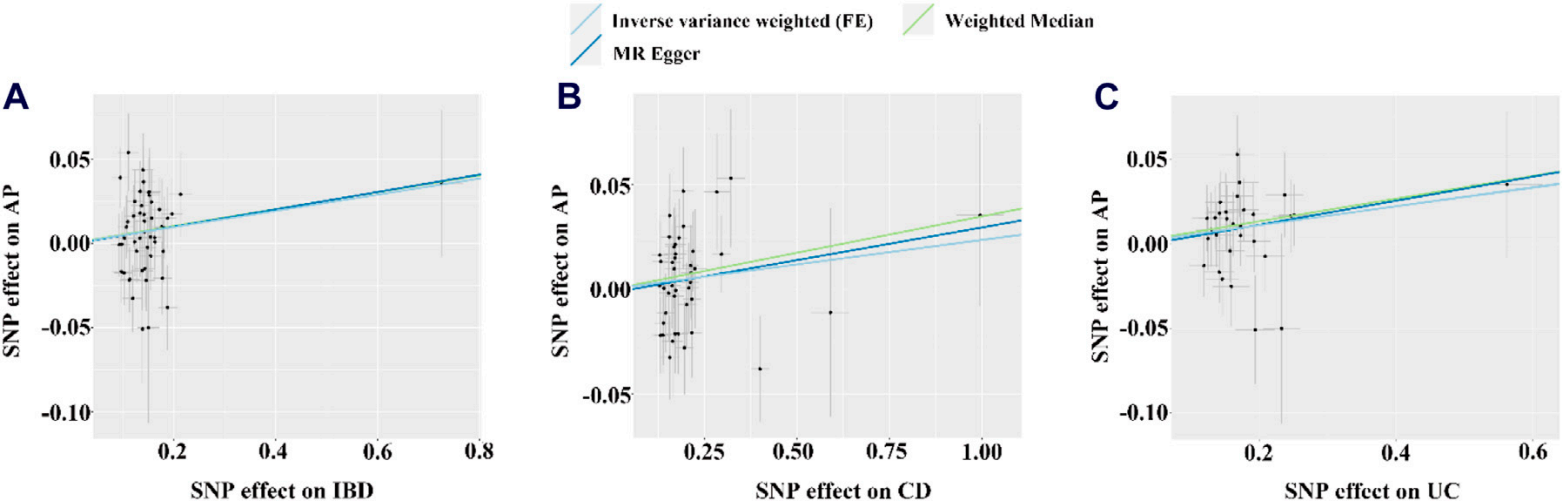
The scatter plots for the causal effect of IBD or its subtypes on AP. **(A–C)** Scatter plot of MR results for IBD, CD and UC as exposure, respectively. FE, fixed effects; the light blue, yellow, and dark blue line on **(A–C)** is represents the fitted regression curve of IVW, MR Egger, and Weighted median method based on the scatter plot, respectively.

Based on the IVW results, we found that IBD (OR = 1.049, 95% CI = 1.010-1.090, *p* = 0.013) and UC (OR = 1.057, 95% CI = 1.013-1.102, *p* = 0.011) were significantly associated with an increased risk of AP (Figure 2; Figures 3A, C). However, we did not find a causal association between CD and AP (OR = 1.023, 95% CI = 0.993-1.055, *p* = 0.134) (Figure 2; Figure 3B). The leave-one-out plot is depicted in Supplementary Figures A–C. The MR Egger pleiotropy test of IBD, CD, and UC showed *p*-values greater than 0.05 (Table 2), and the MR-PRESSO global outlier test of IBD or its subtypes showed no outliers (Table 2). Moreover, the results for IVW-FE, MR-Egger and Weighted median method are consistent in direction (Figure 2). These results indicate that the IVW results in this part are reliable in predicting causal effects.

### 3.3 Genetic IVs for AP

AP was considered as the exposure factor to determine if there is a reverse causality to IBD or its subtypes. We identified 5 IVs in AP (Table 1; Supplementary Table S4), and the F-value range of these IVs is distributed between 10.129 and 11.949 (Table 1). The *p*-value for the Cochran Q test of IBD, CD, and UC studies are 0.985, 0.594, and 0.623, respectively (Table 2). Therefore, we used the fixed-effect model of IVW to evaluate the causal effects of AP on IBD or its subtypes.

### 3.4 Causal effects of acute pancreatitis on IBD or its subtypes

**FIGURE 4:**
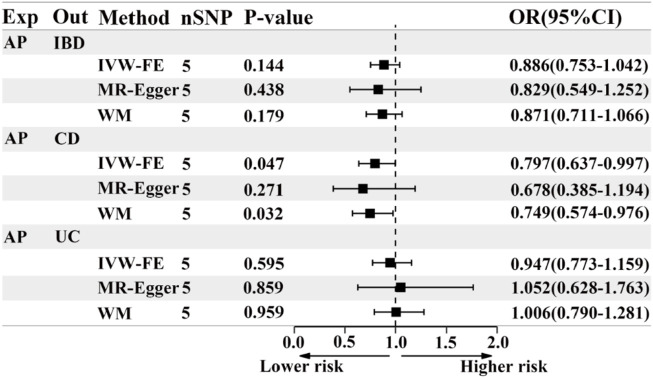
The forest plot of MR results when AP is the exposure and IBD or its subtypes are the outcome. Exp, exposure; Out, outcome; IBD, inflammatory bowel disease; UC, ulcerative colitis; CD, Crohn’s disease; AP, acute pancreatitis; nSNP, the number of instrumental variables, OR, odds ratio; 95%CI, 95% confidence interval; IVW-FE, Inverse-variance weighted (fixed effects); WM, Weighted median.

**FIGURE 5:**
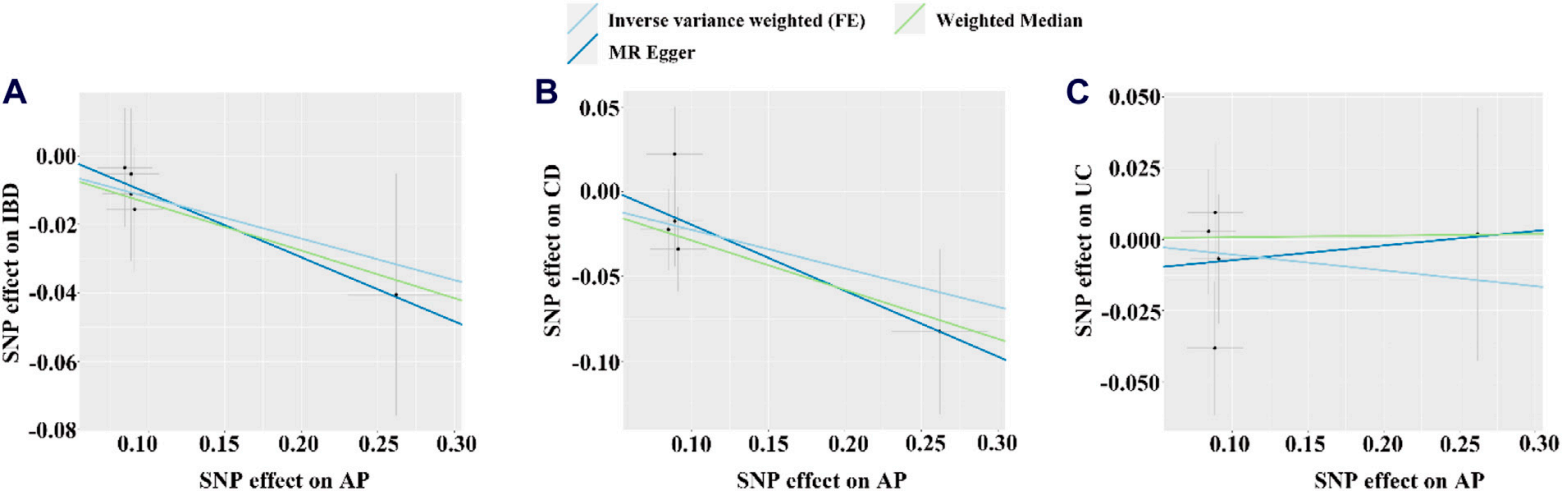
The scatter plot for the causal effect of AP on IBD or its subtypes. **(A–C)** Scatter plot of MR results for IBD, CD and UC as outcome, respectively. FE, fixed effects; the light blue, yellow, and dark blue line on **(A–C)** is represents the fitted regression curve of IVW, MR Egger, and Weighted median method based on the scatter plot, respectively.

Based on the IVW results, we found suggestive evidence that AP was may be associated with a decreased risk of CD (OR = 0.797, 95% CI = 0.637–0.997, *p* = 0.047) (Figure 4; Figure 5B). However, there was no causal association between AP and the risk of IBD (OR = 0.886, 95% CI = 0.753–1.042, *p* = 0.144) or UC (OR = 0.947, 95% CI = 0.773–1.159, *p* = 0.595) (Figure 4; Figures 5A, C). The MR Egger pleiotropy test of IBD, CD, and UC studies yielded *p*-values of 0.754, 0.584, and 0.692, respectively (Table 2). The leave-one-out plot is depicted in Supplementary Figures D–F. Additionally, the MR-PRESSO global outlier test of IBD or its subtypes studies showed no outliers (Table 2). It is worth noting that the results of IBD and CD using IVW-FE, MR-Egger and Weighted median method are consistent in direction, while the results of UC are not (Figure 4).

## 4 Discussion

In this study, we used a two-sample bidirectional MR study to initially assess the potential causal association between IBD or its subtypes with AP. The findings showed that IBD and UC are associated with an increased risk of AP, whereas CD does not causally affect AP. Conversely, AP may be associated with a lower risk of CD but does not causally affect IBD or UC. As we all know, the risk of AP is higher in the IBD population compared to the general population. However, previous studies have mainly relied on observational research (Rasmussen et al., 1999; Munk et al., 2004; Chen et al., 2016), which is susceptible to reverse causality and confounding factors, leaving the causal relationship between the two diseases incompletely established. Traditional randomized controlled trials (RCTs), commonly employed for investigating causal relationships between diseases, frequently encounter ethical, economic, and temporal limitations, hindering their practicality (Nitsch et al., 2006). Unlike traditional RCTs, MR studies use genetic variation as an instrumental variable to study causal relationship, greatly reduce the ethical and economic issues (Davey Smith and Hemani, 2014). In addition, the availability of large-scale public GWAS data has facilitated the establishment of causal relationships between various diseases using MR study. Therefore, our study used Mendelian randomization method to detect the causal relationship between IBD and AP, and the results are more reliable than the previous observational study.

Previous study have shown that the annual incidence rate of AP in the general population ranges from 10 to 44 per 100,000 individuals (Spanier et al., 2008). In addition, a population-based study conducted in Taiwan found that the overall incidence rate of AP in the IBD population was 3.56 times higher than in the general population (31.8 per 10,000 person-years vs. 8.91 per 10,000 person-years) (Chen et al., 2016). Besides, the annual incidence rate of AP in patients with UC was 152.9 per 100,000 individuals (Kim et al., 2017). Moreover, a 10-year follow-up study on CD reported an occurrence rate of AP as 1.4% (Jasdanwala and Babyatsky, 2015). Furthermore, a study conducted on the Danish population found that the risk of AP was 4.3 times higher in CD patients and 2.1 times higher in UC patients compared to the general population (Rasmussen et al., 1999). Meta-analyses have also shown that the risk of pancreatitis in IBD, CD, and UC patients is 2.78, 3.62, and 2.24 times higher, respectively, than in the general population (Pedersen et al., 2020). It should be noted that these results obtained from observational studies cannot establish a causal relationship between AP, and IBD or its subtypes.

Recently, a unidirectional MR analysis demonstrated that IBD increases the risk of AP (Mao et al., 2023). Nevertheless, this study only focuses on the IBD but not its subtypes, and it is unidirectional, which may cause bias. Similarly, our study confirmed that IBD and UC can increase the risk of AP. However, our research found that CD does not directly increase the risk of AP, which seems inconsistent with the high incidence of AP in CD patients found in previous observational studies. This contradiction may be due to CD primarily influencing the risk of AP through intermediary factors that can lead to AP such as medication, gallstones, blood lipids, and education level (Mao et al., 2023). In addition, a multicenter clinical study conducted by Garcia de Paredes et al. found a higher incidence of idiopathic acute pancreatitis (excluding cases caused by known factors such as gallstones, medication, alcohol, or ERCP) in UC compared to CD (Garcia Garcia de Paredes et al., 2020). Considering that our study eliminated factors such as gallstones, medication, blood lipids, education level, etc., as interference, our results are closer to the findings of studies on idiopathic acute pancreatitis (IAP) mentioned above, including that UC can directly influence pancreatitis, while CD primarily affects it through intermediary factors. In summary, our study suggests that the increased risk of AP in IBD is primarily attributed to UC, rather than CD.

Our study is a bidirectional MR study, so we also investigated the role of AP in IBD or its subtypes. Our research found that AP has suggestive evidence of a decreased risk of CD but no causal association with the risk of IBD or UC. Previous observational studies have shown that patients with AP do not have an increased risk of UC compared to the general population, but they do have a significantly increased risk of CD (Li et al., 2020). It should be noted that our results also confirm that the IBD or UC population may have an increased risk of AP, rather than the reverse. However, the results for CD in this study contradict observational studies, and there could be two possible reasons: 1). We relaxed the criteria for selecting IVs (*p* < 5 × 10^−6^) in the AP to IBD MR study, which included some weakly correlated IVs that influenced the final results; 2). The *p*-value (0.047) of the IVW method does not have significant statistical meaning but represents a threshold close to suggestive evidence. Therefore, considering the coexistence of AP and CD in the clinic, the result that CD can reduce the risk of AP should be considered unreliable.

This study performed genetic screening to identify and remove variations associated with confounding factors (such as alcohol consumption, smoking, gallstones, educational level, triglycerides, etc.) that could potentially contribute to AP. Hence, our findings suggest a direct causal influence of UC, rather than CD, on AP. It is worth noting that although CD and UC are both subtypes of IBD, there are differences in their genetic susceptibility loci, which may be the reason for their different effects on AP. The underlying mechanism by which UC contributes to AP is likely related to aberrant immune activation and changes in the intestinal microbiota. Firstly, UC can trigger dysregulated immune system activation, resulting in an excessive production of inflammatory factors (such as TNF-α, IL-1β, IL-6), all of which significantly contribute to the development of AP (Sendler et al., 2013; Wang et al., 2020). Secondly, UC leads to alterations in the intestinal microbiota and intestinal permeability, which subsequently influence the development of AP *via* the gut-pancreas axis (Schepis et al., 2021; Qi-Xiang et al., 2022; Wu et al., 2023). Moreover, the abnormal activation of immune responses not only induces changes in the gut microbiota but also enhances their interaction, thereby exacerbating the incidence of AP (Qi-Xiang et al., 2022; Glaubitz et al., 2023). Therefore, in our clinical practice, if UC patients experience persistent abdominal pain and elevated amylase levels, they should be alert to the occurrence of pancreatitis. In the future, in order to further study the mechanism of the increased risk of AP caused by UC, we will use transcriptome combined with single-cell sequencing and other multi omics methods to elaborate in detail.

Our study has several limitations. Firstly, the *p*-value used for screening when using AP as the exposure factor and IBD or its subtypes as the outcomes is 5 × 10^−6^, which could introduce bias to the results. Secondly, the study focuses on exclusively European populations, so further research is needed to determine if the findings can be generalized to other populations. Thirdly, the PhenoScanner website was used in this study to exclude SNPs that may be associated with confounding factors, aiming to minimize the impact of confounding factors and horizontal pleiotropy. However, given the limited understanding of the biological functions of many SNPs, this method cannot completely eliminate the issue of horizontal pleiotropy in our study. Fourthly, the overlapping participants between the exposure and outcome studies in the two-sample MR analysis cannot be fully evaluated, which may cause some bias. Fifthly, due to the lack of classification for AP in the database, we are unable to evaluate the causal relationship between the specific subtypes of AP and IBD. Lastly, although our results did not indicate a causal effect of AP on UC, the leave-one-out method and the inconsistent results of different MR methods indicated that these results in this situation may not be robust. In the future, as GWAS data continues to advance, we will conduct higher-quality MR studies.

## 5 Conclusion

In conclusion, this study provides genetic evidence that demonstrates a causal influence of IBD (specifically UC) on acute pancreatitis (AP), and CD does not seem to have a causal impact on AP. Further understanding of the intestinal-pancreatic axes may improve our comprehension of this relationship. However, additional research is required to clarify the pathophysiological mechanisms underlying the pathological connection between UC and AP.

## Data Availability

Publicly available datasets were analyzed in this study. This data can be found here: The genome-wide association studies (GWAS) data for IBD, including CD and UC, was obtained from the IEU GWAS database (https://gwas.mrcieu.ac.uk/). The GWAS data for AP was obtained from the FinnGen consortium data (R9 release, https://www.finngen.fi/en).
