# Supplementary material for: Causal association between inflammatory bowel disease and acute pancreatitis: a two-sample bidirectional mendelian randomization study
Source: Front Genet. 2024 Aug 14;15:1324893. doi: 10.3389/fgene.2024.1324893 (PMC11349681; doi:10.3389/fgene.2024.1324893)

Supplementary Figure Legend

The Leave-one-out results of MR study. (A-C) The Leave-one-out plot for IBD, CD and UC as exposure, respectively; (D-F) The Leave-one-out plot for IBD, CD and UC as outcome, respectively. IBD, inflammatory bowel disease; UC, ulcerative colitis; CD, Crohn’s disease; MR, Mendelian randomization.

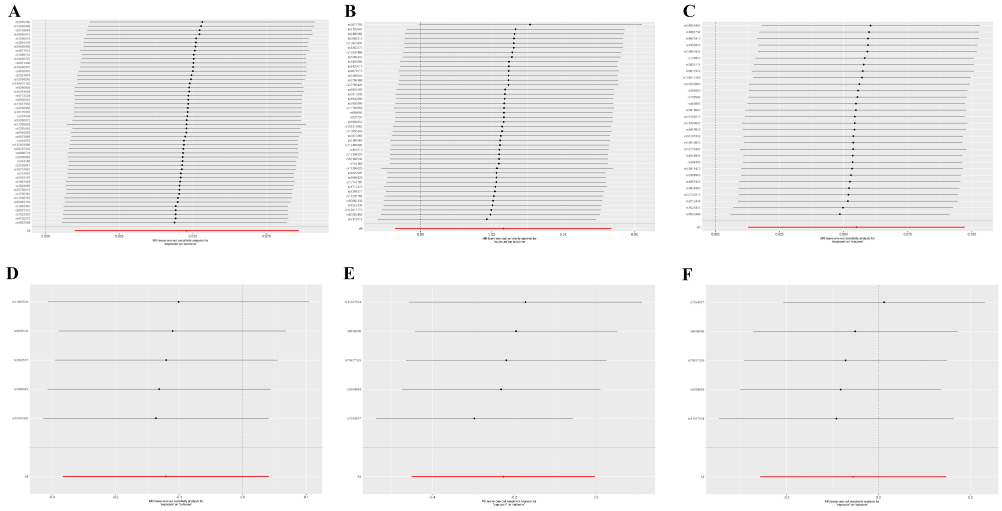

Supplement: Supplementary file 1 [file DataSheet1.PDF]
